# A Mobile Health Intervention for Mental Health Promotion Among University Students: Randomized Controlled Trial

**DOI:** 10.2196/17208

**Published:** 2020-03-20

**Authors:** Marcus Bendtsen, Ulrika Müssener, Catharina Linderoth, Kristin Thomas

**Affiliations:** 1 Linköping University Linköping Sweden

**Keywords:** mHealth, positive mental health, university students, randomized controlled trial

## Abstract

**Background:**

High positive mental health, including the ability to cope with the normal stresses of life, work productively, and be able to contribute to one’s community, has been associated with various health outcomes. The role of positive mental health is therefore increasingly recognized in national mental health promotion programs and policies. Mobile health (mHealth) interventions could be a cost-effective way to disseminate positive psychological interventions to the general population.

**Objective:**

The aim of this study was to estimate the effect of a fully automated mHealth intervention on positive mental health, and anxiety and depression symptomology among Swedish university students using a randomized controlled trial design.

**Methods:**

A 2-arm, single-blind (researchers), parallel-groups randomized controlled trial with an mHealth positive psychology program intervention group and a relevant online mental health information control group was employed to estimate the effect of the novel intervention. Participants were recruited using digital advertising through student health care centers in Sweden. Inclusion criteria were (1) university students, (2) able to read and understand Swedish, (3) and have access to a mobile phone. Exclusion criteria were high positive mental health, as assessed by the Mental Health Continuum Short Form (MHC-SF), or high depression and anxiety symptomology, as assessed by the Hospital Anxiety Depression Scale (HADS). The primary outcome was positive mental health (MHC-SF), and the secondary outcomes were depression and anxiety symptomatology (HADS). The subscales of MHC-SF were also analyzed as exploratory outcomes. Outcomes were measured 3 months after randomization through questionnaires completed on the participants’ mobile phones.

**Results:**

A total of 654 participants (median age 25 years), including 510 (78.0%) identifying as female, were randomized to either the intervention (n=348) or control group (n=306). At follow-up, positive mental health was significantly higher in the intervention group compared with the control group (incidence rate ratio [IRR]=1.067, 95% CI 1.024-1.112, *P*=.002). For both depression and anxiety symptomatology, the intervention group showed significantly lower scores at follow-up compared with the control group (depression: IRR=0.820, 95% CI 0.714-0.942, *P*=.005; anxiety: IRR=0.899, 95% CI 0.840-0.962, *P*=.002). Follow-up rates were lower than expected (58.3% for primary outcomes and 52.3% for secondary outcomes); however, attrition analyses did not identify any systematic attrition with respect to baseline variables.

**Conclusions:**

The mHealth intervention was estimated to be superior to usual care in increasing positive mental health among university students. A protective effect of the intervention was also found on depressive and anxiety symptoms. These findings demonstrate the feasibility of using an automated mobile phone format to enhance positive mental health, which offers promise for the use of mHealth solutions in public mental health promotion.

**Trial Registration:**

International Standard Randomized Controlled Trial Registry ISRCTN54748632; http://www.isrctn.com/ISRCTN54748632

## Introduction

### Background

A substantial body of research has shown a link between high positive mental health and decreased risk of disease [1-5], decreased risk of mental illness [6-9], and increased longevity [3,10]. The promotion of positive mental health among the general population has recently been stressed as the most important goal for the public mental health agenda in Europe [11]. This stems from longitudinal research suggesting a protective effect of positive mental health on mental health problems [9,12].

“Positive mental health” has been defined to encompass feelings of happiness and satisfaction with life (emotional well-being), positive individual functioning regarding self-realization (psychological well-being), and positive societal functioning (social well-being). Furthermore, the two-continua model holds that mental illness and mental health are related but distinct dimensions [13]. The working theory of positive psychology interventions (PPIs) is that elevated positive emotions, thoughts, and behaviors will lead to increased positive mental health [14,15]. PPIs strive to increase the frequency of positive emotions, thoughts, and behaviors through exercises. For instance, to increase positive thinking of gratitude, individuals are asked “to think about three things that you are grateful for today” [5,15]. Although there is evidence to support the efficiency of PPIs on positive mental health for both healthy and clinical populations, more research is needed.

Two meta-analyses of PPIs reported relatively small effect sizes [5,15], which may be due to the composition of the included interventions. Most studies included in the meta-analyses examined the effect of individual exercises targeting only one aspect of positive mental health (eg, using a gratitude journal to increase positive thinking). However, as positive mental health is a multilayered construct [13], a multicomponent intervention taking into account emotional, social, and psychological well-being may be more effective. A systematic review and meta-analysis on multicomponent PPIs found supporting evidence in terms of positive mental health and depression, and potentially anxiety and stress. However, the authors concluded that larger and more rigorous studies are needed to move the research field forward, for instance through sufficiently powered trials with transparent methodological reporting [16]. In addition, a limited number of studies included in the aforementioned reviews were delivered through mobile health (mHealth) interventions, an otherwise fast-growing practice and research field.

mHealth interventions could potentially be a cost-effective means to disseminate PPIs to a large population [17,18]. There are now countless mobile apps commercially available that target positive mental health among the general population; however, the majority of these apps lack experimental evidence, are not theory-based, and have not been scientifically evaluated [19]. A review on the effect of digital interventions (eg, mobile apps) on mental health showed a small to medium effect, suggesting that mental health problems decreased while positive mental health increased among those with access to the interventions. However, the overall quality of the body of evidence in the review was low due to several concerns regarding risk of bias [20]. Another review summarized the evidence for theory-driven and evidence-based mental health eResources (eg, website or mobile apps) and only found one randomized controlled trial, suggesting a lack of valid evidence. The authors concluded that eResources for mental health have the potential to be widely effective, but that more rigorous studies are needed to clarify the benefits [21].

### Objectives

The aim of this study was to estimate the effect of a fully automated mHealth intervention on positive mental health and anxiety and depression symptomology among Swedish university students using a randomized controlled trial design.

The primary hypothesis was that positive mental health will differ among groups at 3 months postrandomization, with those having access to the novel mHealth intervention reporting higher scores on the Mental Health Continuum Short Form (MHC-SF). The secondary hypotheses were: (1) depression and anxiety symptomology will differ among groups at 3 months postrandomization, with those having access to the novel mHealth intervention reporting lower scores on the subscales of the Hospital Anxiety Depression Scale (HADS); and (2) emotional, social, and psychological well-being will differ among groups at 3 months postrandomization, with those having access to the novel mHealth intervention reporting higher scores on the subscales of the MHC-SF.

## Methods

### Trial Design

This trial was prospectively registered with the International Standard Randomized Controlled Trial Registry (ISRCTN54748632) and a trial protocol was made available prior to trial commencement [22]. The study received ethical approval from the Regional Ethical Review Board of Linköping University, Sweden (Dnr. 2018/519-32).

A 2-arm, single-blind (researchers), parallel-groups randomized controlled trial (1:1) was employed to estimate the effect of the novel mHealth intervention. Participants were allocated to either an intervention group (mHealth program) or control group (treatment as usual). Prior to trial commencement, but after trial registration and publication of the protocol, it was decided to remove age restrictions in the eligibility criteria. This was done so that participants would more accurately represent all Swedish university students and not only young adults.

### Participants

Inclusion criteria were (1) university students, (2) able to read and understand Swedish, (3) and have access to a mobile phone. The exclusion criterion was high positive mental health defined as a score of 70 or more on the MHC-SF [23]. As the intervention was not designed to treat mental health problems, a second exclusion criterion was depression and anxiety symptomatology defined as a score of greater than or equal to 10 on both subscales of the HADS [24]. Individuals excluded due to a high HADS score were encouraged to seek help and were provided information on where to receive support (contact information of their local student health center, primary care center, or governmental national health website).

Recruitment of students was carried out at 15 universities in Sweden, which lasted between October 8, 2018 and April 30, 2019. Recruitment was achieved through digital advertising, including email, university websites, student health care center websites, and learning management systems used by the universities. The advertisement included information on the study aims, confidentiality, and trial design. Students indicated their interest in taking part in the trial by texting a dedicated telephone number included in the advertisement material. The students then received a text message response with a link to the informed consent form and completed an online baseline questionnaire on their mobile phones. Eligibility was determined from responses to the baseline questionnaire, and eligible participants were automatically randomized to either the intervention or control group. Participants were given information on which group they had been allocated to.

### Intervention

The intervention was a fully automated mHealth positive psychology multicomponent program. The program was based on theories and empirical evidence from the positive psychology research field [13,25] and aimed to enhance users’ positive mental health. The program encompassed information about well-being, validated self-help exercises, brief tips, self-monitoring, and personalized feedback. Text messages were sent to users throughout the program, with an average of one text message per day, and included text and links to interactive exercises and further reading. The program ran for 10 weeks, with a new theme introduced each week. The themes used have been shown to be important for positive mental health, and included gratitude, savoring, positive emotions, personal strengths, positive relations, social environment, health behaviors, optimism, and goal setting. In the final week, users were recommended to reflect on the program, for instance by writing down any lessons learnt. Details of the intervention can be found elsewhere (Multimedia Appendix 1) [22].

Participants allocated to the control group were informed of their allocation status via a text message. The text message included contact details to their local student health center, primary care center, and governmental national health website. At the time of the trial, this was considered treatment as usual.

### Outcomes

As the primary outcome, positive mental health was measured using the 14-item MHC-SF [23], in which higher scores indicate greater emotional, social, and psychological well-being (range 0-84). Secondary outcomes included depression and anxiety symptomatology, measured as the score on corresponding subscales of the HADS [24]. Each subscale consists of 7 items for a total of 14 items. Item scores are calculated into a total scale score for anxiety (range 0-21) and depression (range 0-21), with higher scores indicating higher depression and anxiety. We further measured emotional, social, and psychological well-being as exploratory outcomes based on the mean score of each subscale of the MHC-SF.

A SPIRIT [26] checklist depicting study procedures and measurements is presented in Table 1. At baseline, the participants’ age, gender, and social status were recorded, along with primary, secondary, and exploratory outcomes. Two face-valid mediator items, aimed at measuring the frequency of positive thoughts and emotions, were also determined at baseline according to responses to the following questions: ‘‘During the last week, to what extent have you experienced positive thoughts?” and “During the last week, to what extent have you experienced positive emotions?” Participants were asked to rate their response on a scale ranging from 1 (‘‘not at all’’) to 10 (‘‘to a very high extent’’).

Five weeks after randomization, participants were sent a text message with a link to the two mediator items. Three months after randomization, participants were sent a text message with a link to the follow-up questionnaire. The follow-up questionnaire explored primary, secondary, and exploratory outcomes (MHC-SF and HADS).

Participants that did not respond to the initial follow-up attempt were sent up to 4 reminders 2 days apart. Those who had still not responded were called by telephone (maximum 3 call attempts).

**Table 1:** SPIRIT checklist depicting study procedures, measurements, and timeline.

		Study Period					
		Enrollment	Allocation	Post-allocation		Follow-up	
			0	Week 5	Week 10	3 months	
**Enrollment**							
	Eligibility screen		X				
	Informed consent	X					
**Interventions**							
	Text message intervention		X	X	X		
	Referred to sources of mental health information		X	X	X		
**Assessments**							
	Baseline demographics		X				
	MHC-SF^a^		X			X	
	Mediators		X	X		X	
	HADS^b^		X			X	

^a^MHC-SF: Mental Health Continuum Short Form.

^b^HADS: Hospital Anxiety Depression Scale.

### Sample Size

A power analysis was conducted to determine the necessary number of participants to recruit for the study. To detect a standardized effect size of 0.3, the average score in the intervention group should exceed scores of 62% of the control group; hence, a total of 352 participants was expected to be required. The calculations were performed assuming an 80% chance of detecting a difference at a significance level of alpha=.05 (two-tailed). Assuming that 70% of the participants would respond to the follow-up questionnaire, it was deemed necessary to recruit a total of 503 participants.

### Randomization and Blinding

After completing the online baseline questionnaire, participants were randomly allocated a number 0 or 1 with equal probability using Java’s built-in random number generator (java.util. Random). Participants with a 0 were allocated to the control group, and participants with a 1 were allocated to the intervention group. Generation of the randomization sequence was therefore fully computerized, and allocation was concealed from participants and research team members.

### Statistical Analysis

All analyses conformed to the prespecified statistical analysis plan in the trial protocol [22]. For Hypothesis 1, the primary outcome (score of the MHC-SF) was analyzed. This score represents a discrete measure that may be skewed; thus, we regressed this outcome on group allocation and baseline variables using negative binomial regression. Hypothesis 2 was investigated by analyzing secondary outcome measures (subscales of the HADS). This score is also a discrete measure that may be skewed, and was regressed against group allocation and baseline variables using negative binomial regression. Hypothesis 3 was investigated by analyzing the subscales of the MHC-SF: emotional well-being, social well-being, and psychological well-being. These are mean scores from Likert scale items, which should tend toward normality owing to the law of large numbers. Therefore, we regressed the individual scores against group allocation and baseline variables using normal linear regression.

### Analytical Approaches

Analyses were performed under the intention-to-treat principle, including all randomized individuals, and no imputations were made for missing values. Missing outcome data were initially handled by a complete-case analysis, which assumed that data were missing completely at random (MCAR). If data are systematically missing, this may indicate that early responders differ from late responders, and in extension that late responders are more similar to nonresponders. We therefore explored the plausibility of the MCAR assumption by regressing the primary outcomes on the number of follow-up attempts needed before a response was recorded. To further explore the MCAR assumption, attrition was investigated among study groups by comparing baseline characteristics between those who did and did not respond to the follow-up questionnaire.

Both adjusted and unadjusted models were designed when investigating all hypotheses; however, it was decided a priori to primarily use adjusted models [22]. For all models, the coefficients of interest were assessed for statistical significance using a null hypothesis testing approach, where all tests were two-tailed at a significance level of alpha=.05.

Effect modification tests were performed to assess if any of the baseline characteristics moderated the effect of the intervention.

## Results

### Recruitment

**Figure 1:**
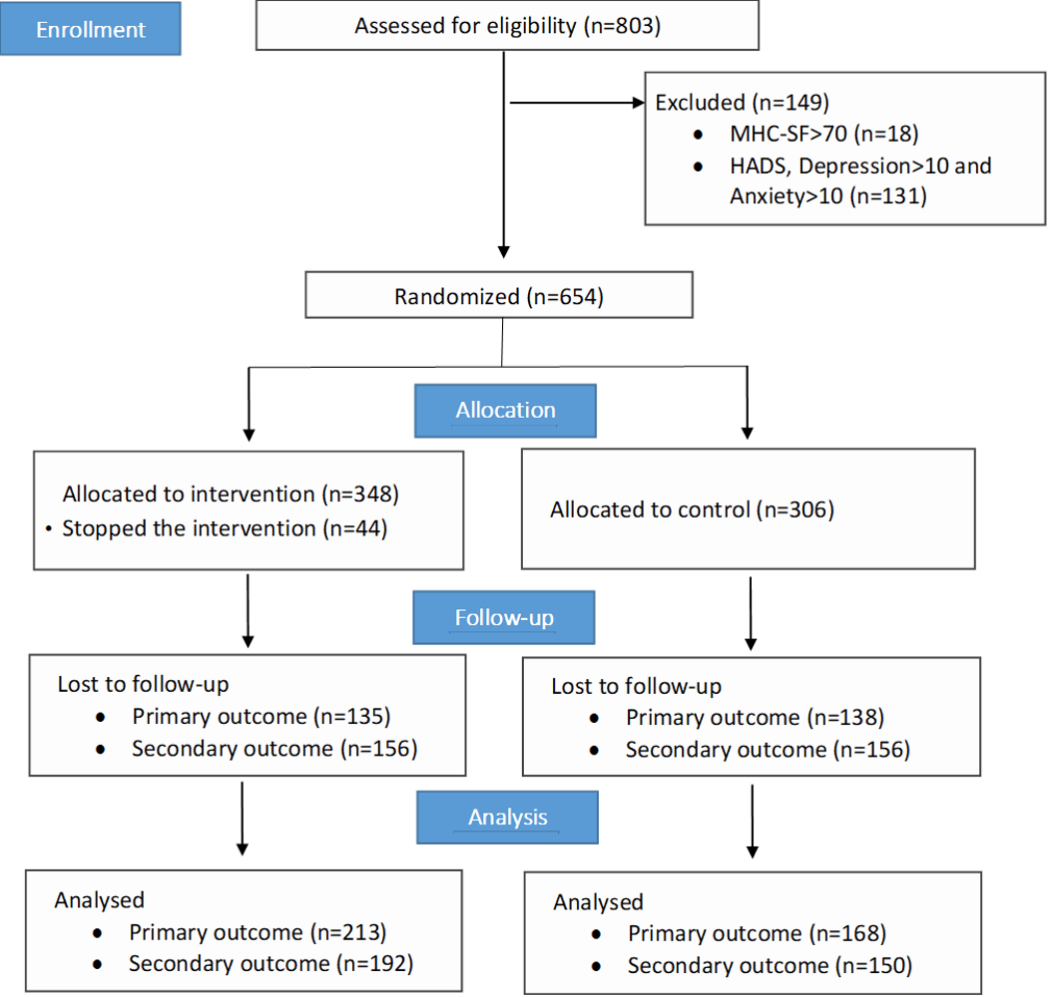
CONSORT diagram of trial participant flow. HADS: Hospital Anxiety Depression Scale; MHC-SF: Mental Health Continuum Short Form.

Figure 1 depicts a CONSORT diagram of the trial participant flow. A total of 654 participants were randomized: 348 (53.2%) to the intervention group and 306 (46.8%) to the control group. In the intervention group, 340 (79.2%) participants received all messages, and the remaining 44 individuals decided to stop the intervention before completion. All participants were contacted at follow-up regardless of adherence to the intervention. A total of 381 (58.3%) participants were included in the analysis of the primary outcomes, and 342 (52.3%) were included in the analysis of secondary outcomes. No participants explicitly requested to be removed from the trial.

### Baseline Data

Table 2 summarizes the baseline characteristics of the recruited participants. There were no statistically significantly differences between the intervention and control groups with respect to any of the measured baseline characteristics. Notably, the great majority of participants identified as female.

**Table 2:** Baseline characteristics for both study groups.

Variable		Intervention (n=348)	Control (n=306)	*P* value
**Gender, n (%)**				.42^a^
	Female	277 (79.6)	233 (76.1)	
	Male	69 (19.8)	69 (22.5)	
	Other	2 (0.6)	4 (1.3)	
Age (years), median (IQR^b^)		25 (22-29)	26 (22-30)	.28^c^
**Marital status, n (%)**				.73^a^
	No partner; no children at home	170 (48.9)	149 (48.7)	
	No partner; children at home	7 (2.0)	6 (2.0)	
	Partner; no children at home	95 (27.3)	72 (23.5)	
	Partner; children at home	46 (13.2)	47 (15.4)	
	Partner but not living together	30 (8.6)	32 (10.5)	
Positive thoughts, median (IQR)		6 (4-7)	6 (4-7)	.43^c^
Positive emotions, median (IQR)		5 (4-7)	5 (4-7)	.80^c^
Anxiety^d^, median (IQR)		12 (10-15)	12 (10-14)	.58^c^
Depression^e^, median (IQR)		6 (4-8)	6 (4-8)	.93^c^
Total well-being^e^, median (IQR)		47.5 (40-55)	50 (40.25-57)	.14^c^
Emotional well-being^f^, mean (SD)		3.79 (0.89)	3.77 (0.92)	.78^g^
Social well-being^f^, mean (SD)		2.93 (0.92)	3.06 (0.89)	.07^g^
Psychological well-being^f^, mean (SD)		3.59 (0.91)	3.67 (0.87)	.24^g^

^a^Fisher exact test.

^b^IQR: interquartile range.

^c^Mann-Whitney *U* test.

^d^Hospital Anxiety Depression Scale.

^e^Mental Health Continuum Short Form.

^f^Subscales of the Mental Health Continuum Short Form calculated as means of responses to each respective subset of questions.

^g^Student *t* test.

### Outcomes

At 3 months after randomization, primary outcome data were collected from 213 (61.2%) participants in the intervention group and 168 (54.9%) participants in the control group. Secondary outcome data were collected from 192 (55.2%) participants in the intervention group and from 150 (49.0%) participants in the control group. These data were used to investigate the trial hypotheses according to the statistical analysis plan. The results are summarized in Table 3.

At the 3-month follow-up, positive mental health measured by the MHC-SF was significantly higher in the intervention group compared with that of the control group, which supported Hypothesis 1. Cronbach alpha was .91 including all items of MHC-SF, indicating high reliability of the measure. In addition, the scores of depression and anxiety symptoms (subscales of HADS) were both significantly lower in the intervention group compared with those of the control group, supporting Hypothesis 2. Cronbach alpha for the items included in the anxiety subscale was .81, and was .83 for the items included in the depression subscale, indicating high reliability of both measures. The subscales of the MHC-SF were all significantly higher in the intervention group compared with those of the control group. Emotional well-being, social well-being, and psychological well-being scores were all in the anticipated direction supporting Hypothesis 3. Cronbach alpha indicated high reliability for all three subscales at .83, .79, and .85 for emotional, social, and psychological well-being, respectively.

**Table 3:** Summary of trial hypotheses analyses at the 3-month follow-up assessment.

Outcome			Follow-up		Difference^a^	Unadjusted model				Adjusted model^b^				
						Regression coefficient^c^ (95% CI)		*P* value			Regression coefficient (95% CI)		*P* value	
**Primary outcome**														
	**Total well-being, median (IQR^d^)**													
		Intervention (n=213)		56 (47 to 65)	7 (1 to 13)		1.035 (0.985-1.087)		.17			1.067 (1.024-1.112)		.002
		Control (n=168)		56 (42 to 64.25)	3 (–4 to 10.25)									
**Secondary outcomes**														
	**Depression, median (IQR)**													
		Intervention (n=192)		4 (2 to 6)	–2 (–4 to 0)		0.817 (0.699-0.954)		.01			0.820 (0.714-0.942)		.005
		Control (n=150)		4 (2 to 8)	–1 (–3 to –1)									
	**Anxiety, median (IQR)**													
		Intervention (n=192)		9 (7 to 12)	–2 (–4.25 to 0)		0.926 (0.854-1.004)		.06			0.899 (0.840-0.962)		.002
		Control (n=150)		10 (8 to 13)	–1 (–3 to –1)									
**Exploratory outcomes**														
	**Emotional well-being, mean (SD)**													
		Intervention (n=213)		4.23 (0.88)	0.45 (0.86)		0.152 (–0.037-0.341)		.11			0.222 (0.062-0.383)		.007
		Control (n=168)		4.08 (0.99)	0.20 (0.93)									
	**Social well-being, mean (SD)**													
		Intervention (n=213)		3.47 (1.03)	0.51 (0.89)		0.082 (–0.136-0.300)		.46			0.203 (0.021-0.385)		.03
		Control (n=168)		3.39 (1.13)	0.25 (0.98)									
	**Psychological well-being, mean (SD)**													
		Intervention (n=213)		4.20 (0.99)	0.60 (0.90)		0.166 (–0.040-0.372)		.11			0.272 (0.093-0.451)		.003
		Control (n=168)		4.04 (1.05)	0.28 (0.99)									

^a^Difference between follow-up measurement and baseline measurement.

^b^Adjusted for outcome measure, gender, age, marital status, and mediators.

^c^Incidence rate ratio for group by negative binomial regression for total well-being, depression, and anxiety; linear coefficient for emotional well-being, social well-being, and psychological well-being.

^d^IQR: interquartile range.

### Sensitivity Analyses

Sensitivity analyses were carried out for the primary and secondary hypotheses in which the missing outcomes were set to baseline values. No large differences in direction or statistical significance were found for the three analyses (total well-being incidence rate ratio [IRR]=1.052, 95% CI 1.024-1.08, *P*<.001; depression IRR=0.878, 95% CI 0.817-0.943, *P*<.001; anxiety IRR=0.936, 95% CI 0.894-0.981, *P*=.006).

### Effect Modification Analyses

Effect modification models were explored for the primary outcome. All variables measured at baseline were considered in separate models, and were compared with the noninteraction model using likelihood ratio tests. There was weak evidence for an interaction effect with respect to age (*P*=.03) and marital status (*P*=.03).

**Figure 2:**
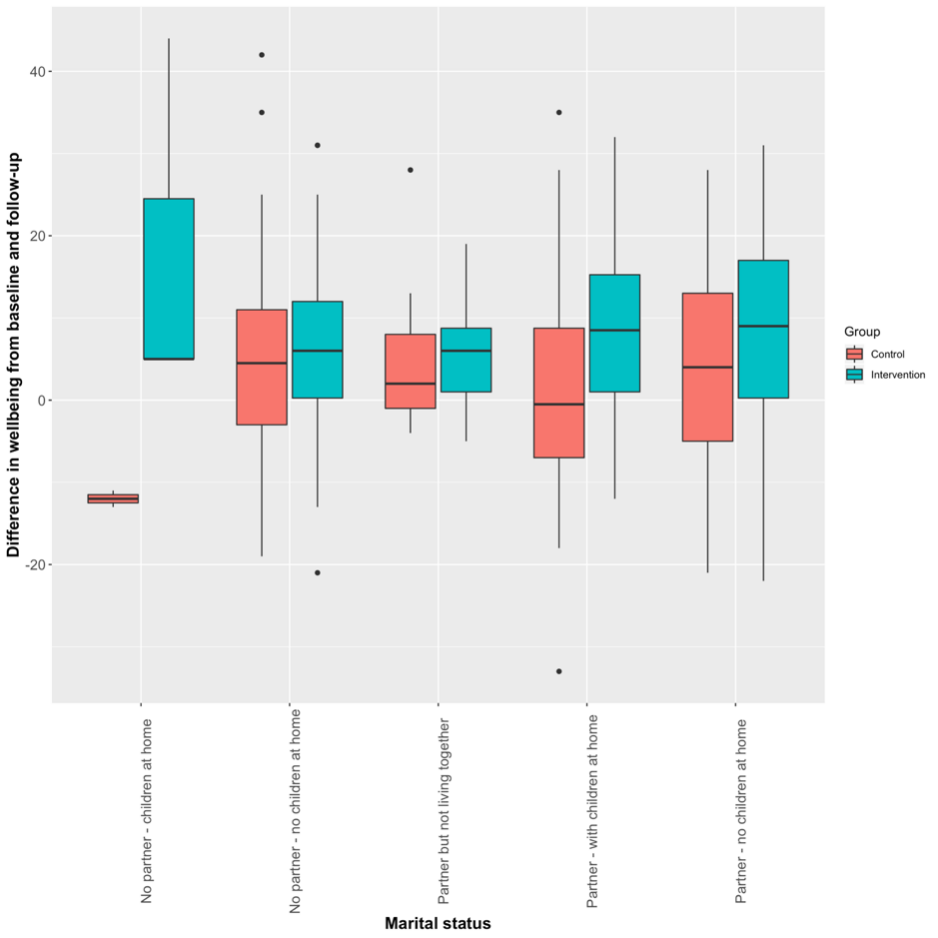
Interaction between group allocation and marital status.

Exploring the interaction with marital status through a box plot (Figure 2) revealed that it was the marital status category “No partner; children at home” that was driving the modification effect. Among the participants analyzed, only 5 responded with this option, and removing them from the analysis removed the interaction effect for both marital status and age. Overall, any interaction between group allocation, age, and marital status was not considered to be strongly supported by the data.

### Attrition Analyses

We used two approaches to explore the MCAR assumption: (1) response/nonresponse at follow-up was regressed against baseline characteristics, and (2) primary outcome was regressed against the number of attempts required to reach the respondent to collect follow-up data. The second approach assumes that nonresponders are actually late responders; thus, if there is an association between follow-up attempts with primary outcome, then nonresponders may be systematically different from responders. However, there was no significant association between number of follow-up attempts and response, suggesting that there was no systematic difference between early and late responders.

**Figure 3:**
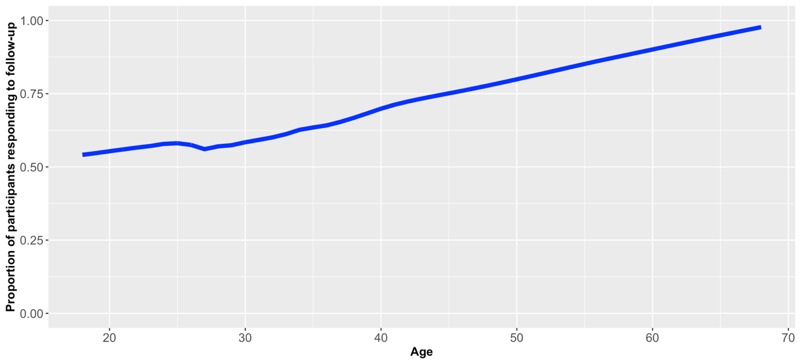
Locally weighted scatterplot smoothing: age against proportion of participants responding to follow-up for all participants.

**Figure 4:**
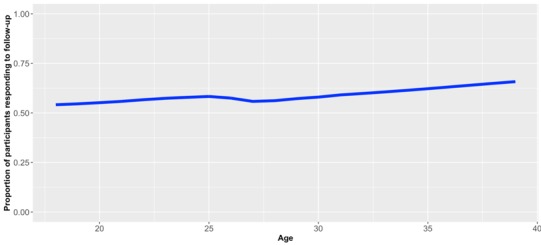
Locally weighted scatterplot smoothing: age against proportion of participants responding to follow-up, including only participants aged less than 40 years at baseline.

When regressing response/nonresponse against baseline characteristics, we found that age was potentially associated with response, as the odds ratio was 1.03 (95% CI 1.008-1.053, *P*=.008), suggesting that older participants were more likely to respond to follow-up. Locally weighted scatterplot smoothing confirmed this association (Figure 3), which was mainly driven by participants aged 40 or more, 40/54 (74.1%) of whom responded to follow-up, compared to 338/597 (56.6%) among those under 40 years of age. No such association between age and response was observed among participants under 40 years old (Figure 4).

## Discussion

### Principal Findings

We examined whether a fully automated mHealth intervention was effective in increasing positive mental health among university students. The results suggested that the intervention may be superior to usual care in increasing positive mental health for this group (MHC-SF: IRR 1.067, 95% CI 1.024-1.112, *P*=.002). In addition, the results indicated a protective effect of the intervention on depressive and anxiety symptoms.

In general, our findings confirm previous research on the effect of PPIs on mental health. Reviews investigating PPIs delivered via face-to-face and self-help (not mHealth) modalities have shown an increase in positive mental health and enabled individuals to manage mental health problems (eg, based on decreased anxiety and worry scores) [5,15]. Interestingly, studies using digital-based interventions (eg, DVD, VCR, or Web-based) showed a comparable effect on mental health, supporting the viability of digital interventions in this area [27,28]. For example, a recent study found that an intervention encompassing self-help book and email support successfully increased positive mental health and decreased depression among adults [29]. Our findings add to this growing body of evidence, and further demonstrate the effect and feasibility of delivering PPIs in a fully automated mobile phone format.

Furthermore, our findings show that a relatively inclusive program (10 weeks including 9 positive psychology themes) was acceptable for participants to engage with, as a strong majority of participants completed the program. Research on multicomponent PPIs has shown that these integral programs influence positive mental health and depression, and potentially anxiety and stress [16]. Our study adds to this body of research showing that a quite comprehensive program (including several activities that targeted several well-being components) was feasible and acceptable among the target group and was effectively delivered via mobile phones.

Several factors that can moderate the effect of PPIs have been proposed, including the duration of an intervention, baseline affect states of participants (eg, healthy vs subclinical populations), and recruitment methods (self-referral vs referred by a health care practitioner). An early review on PPIs delivered in group or self-administered contexts indicated that the optimal duration of PPIs was about 8 weeks [15]. Our findings provide support for the acceptability and feasibility of longer-duration PPIs. In future research, it would be interesting to investigate further dose-response and person-activity fit aspects of PPIs; that is, the effect of the variety, frequency, and tailoring of activities on mental health outcomes. PPIs tend to be more effective among subclinical populations (eg, moderate anxiety and depressiveness). Along similar lines, although our intervention was developed as a preventative measure targeting the healthy population, the findings indicate a protective effect on anxiety and depressive symptomatology for this group. Furthermore, there is controversy in the literature as to whether PPIs are more effective among self-referral groups or when they are disseminated through health care referral routes; however, self-referral seems to be more effective when considering only healthy populations [4,15]. From a public health perspective, recruitment via self-referral to self-help PPIs can offer cost-effective mental health promotion tools to reach large target groups.

Finally, the participants in our study were mostly women. Although our analyses did not indicate bias regarding gender, the generalizability of the findings could be limited. Previous research investigating PPIs that have used similar recruitment methods (self-selected) reported similar rates (about 85% women) [29]. This could indicate a greater interest and willingness among women to take part in well-being studies. However, as positive mental health does not necessarily differ between men and women, future studies could benefit from identifying recruitment strategies that can reach men to a greater extent.

### Limitations

The trial design does not allow us to isolate specific themes of the program to estimate their individual effects. That is, we cannot identify if a specific theme and its accompanying exercises (eg, practicing gratitude) increased positive mental health to a greater extent than other themes. However, the literature suggests that single-component interventions have smaller effect sizes compared to multicomponent interventions [5,29], indicating that it would be more effective to invest in the latter to increase well-being among the general population. Future studies with alternative designs are needed to investigate how different components of the program, or combination of components, contribute to mental health outcomes.

A prominent limitation of this trial is the risk of attrition bias due to low follow-up rates. Despite the lack of strong evidence against MCAR, the assumption cannot be formally tested, and therefore the presented results should be interpreted under this limitation. We designed the trial under the assumption of achieving similar follow-up rates as in previous trials on the same target group (reaching as high as 90% of students [30,31]); however, we could not achieve the same retention rate in this trial. One factor causing this increased attrition was the design of the MHC-SF, which is hard to complete over the telephone given that it is relatively long and difficult to communicate verbally. Although the use of validated instruments such as MCH-SF and HADS is a strength of this trial, future research on PPIs targeting university students should consider other item sets for measuring outcomes to increase retention.

We did not include an active control in this trial, mainly with a view to estimate the total effect of the intervention compared to minimal contact, but also since there are no other available effective interventions that could be offered at this scale and through digital means. There are limitations to this approach, as it makes blinding difficult and may therefore result in performance bias. To reduce the risk of performance bias, we ensured that all participants were treated equally by automating all processes and not having any interaction between the research personnel and participants. However, one factor increasing the risk of detection bias stems from the use of telephone follow-up for nonresponders, as participants may have revealed to the interviewer which group they belonged to. We decided to go ahead with telephone follow-ups as the benefit in terms of reduced attrition bias was believed to outweigh the risk of detection bias, and interviewers were instructed to avoid discussions about group allocation.

Participants in this trial were recruited naturally through passive advertisement in printed and digital media provided by the student health care centers. This closely mimics the way that individuals would come in contact with the intervention had it been disseminated outside the trial setting. Thus, the participants recruited are comparable to participants who would use the intervention in a real-world setting. The results herein can be considered generalizable to a wider context of university students; however, the limitations discussed above should be taken into consideration before any decision for dissemination is made.

### Conclusions

An mHealth intervention based on theories and empirical evidence from the positive psychology research field [16,25] was estimated to have a superior effect on positive mental health compared to usual care (MHC-SF: IRR 1.067, 95% CI 1.024-1.112, *P*=.002). In addition, a protective effect of the intervention was found on depressive and anxiety symptoms. These findings demonstrate the feasibility of using an automated mobile phone format to enhance positive mental health.

Disseminating mHealth PPIs could have significant public health benefits. It has been estimated that even small improvements in positive mental health in the general population could yield large preventive effects on psychopathology [32]. Previous studies have shown that targeting PPIs among people with low to moderate well-being not only increases positive mental health but can also prevent future anxiety and depression [7,8]. Our findings support the notion that mHealth solutions can be used in public mental health promotion.

Future research should focus on estimating the effect of PPI mHealth interventions among other societal groups, including studies with the elderly, minorities, and general population. Dismantling of the content of the mHealth intervention to identify which combinations of themes show the greatest effect can guide future developments of PPIs, such that even more effective interventions may be developed. Finally, creating strategies to implement and disseminate PPI mHealth interventions to a general audience will also be necessary to maximize the societal benefit of these interventions.

## Appendix

Multimedia Appendix 1 Table illustrating the themes of the program, number of text messages per week and examples of content.

Multimedia Appendix 2 CONSORT-eHEALTH checklist (V 1.6.1).

## Abbreviations

HADS: Hospital Anxiety Depression Scale
IRR: incidence rate ratio
MCAR: missing completely at random
MHC-SF: Mental Health Continuum Short Form
mHealth: mobile health
PPI: positive psychology intervention
