# Supplementary material for: A Mobile Health Intervention for Mental Health Promotion Among University Students: Randomized Controlled Trial
Source: JMIR Mhealth Uhealth. 2020 Mar 20;8(3):e17208. doi: 10.2196/17208 (PMC7125436; doi:10.2196/17208)
Supplement: Multimedia Appendix 1 [file mhealth_v8i3e17208_app1.docx]

| **Appendix 1** Table illustrating the themes of the program, number of text messages per week and examples of content. | | | |
| --- | --- | --- | --- |
| **Week** | **Theme** | **Number of text messages** | **Examples of content** |
| 1 | Gratitude | 7 | - Reflect on things you are grateful for in life. - Reflect on positive events. - Information on gratitude. |
| 2 | Savouring | 12 | - Mindfulness exercise that prompted users to use your five senses to savour the moment. - Reminiscing on positive events - Information on savouring and living in the moment. |
| 3 | Thought patterns | 11 | - Strategies to break negative thought patterns - Activities to break negative thought patterns - Information on how our thoughts influence our well-being |
| 4 | Personal strengths | 6 | - Identify personal strengths - Develop/expand personal strengths - Information on personal strengths |
| 5 | Kindness | 9 | - Prompt to do acts of kindness - Reflect on how kindness affect our well-being - Information on kindness |
| 6 | Empathy and meaningful relations | 7 | - Tips on how to strengthen relationships - Tips on communication styles - Information on how our social environment influence our well-being |
| 7 | Health behaviours | 13 | - Meditation exercise - Information on the role of physical activity, eating habits, moderate alcohol consumption and smoking on our well-being. |
| 8 | Optimism and thoughts about the future | 9 | - My best possible self exercise - Strategies of how to handle setbacks - Information on the role of optimism on our well-being |
| 9 | Goal setting | 9 | - Define personal goals - Reflect on personal goals and their meaning |
| 10 | Plan for the future | 9 | - Identify favourite exercise in the program - Lessons learnt - Action planning |
